# Suture Pull-Through Insertion Techniques for Descemet Stripping Automated Endothelial Keratoplasty in Chinese Phakic Eyes: Outcomes and Complications

**DOI:** 10.1371/journal.pone.0061929

**Published:** 2013-04-23

**Authors:** Ying Hong, Rong-mei Peng, Miao Wang, Hong-qiang Qu, Jing Hong

**Affiliations:** 1 Department of Ophthalmology, Peking University Third Hospital, Beijing, China; 2 Capital Institute of Pediatrics, Beijing, China; University of Illinois at Chicago, United States of America

## Abstract

**Purpose:**

To investigate the outcomes and complications of suture pull-through insertion techniques for Descemet stripping automated endothelial keratoplasty (DSAEK) in Chinese phakic eyes.

**Patients and Methods:**

Retrospective case series. Included in the study were all Chinese patients with phakic eyes who underwent DSAEK at Peking University Third Hospital from August 2008 to August 2011. All ocular diseases of the patients were recorded. Distance visual acuity (DVA), near visual acuity (NVA), intraocular pressure (IOP), anterior chamber depth (ACD), central corneal thickness (CCT), and corneal endothelial cell density (ECD) were compared prior to and 12 months after DSAEK. The DSAEK success rate, endothelial cell loss (ECL), complications, and prognosis were analyzed. All patients had at least 12 months of follow up.

**Results:**

Twenty-one eyes of 16 patients were included (11 males and 5 females). Ages ranged from 2 to 47 years with an average age of 29.8 years. The average follow up was 15.4 months (ranging from 12 to 36 months). Diagnoses included 7 eyes (4 patients) with corneal endothelial dystrophy and 14 eyes (12 patients) with bullous keratopathy. Presurgical DVA and NVA (LogMAR) were 1.7±0.7 and 1.2±0.4; postsurgical DVA and NVA were 0.8±0.6 and 0.7±0.5; Z = −3.517, −2.764; P<0.001 and P = 0.006 respectively. Presurgical IOP was 15.8±3.7 mm Hg; postsurgical IOP was 15.2±2.6 mm Hg; Z = −0.505, P = 0.614. Presurgical ACD was 3.00±0.74 mm; postsurgical ACD was 2.72±0.59 mm; Z = −0.524, P = 0.600. Donor ECD was 2992±163 cells/mm^2^, ECD was 1836±412 cells/mm^2^ with a 12-month postsurgical ECL of 39%. Success rate was 86%. Surgery complications included pupillary block-induced hypertension in 5 eyes (24%), graft detachment in 3 eyes (14%), and graft dislocation in 1 eye (5%).

**Conclusions:**

DSAEK on Chinese phakic eyes can significantly improve DVA and NVA by preserving the patient’s own crystalline lens. DSAEK is an optional surgery for patients who need to preserve accommodative function. More attention should be given to postsurgical pupillary block-induced hypertension.

## Introduction

Recent research shows that Descemet stripping automated endothelial keratoplasty (DSAEK) has replaced penetrating keratoplasty (PKP) as the standard surgical procedure for corneal endothelial disease.[1]–[3] DSAEK is a safer procedure than PKP with a lower rejection rate, less induced astigmatism, and a more rapid recovery [1], [4]–[6].

DSAEK needs enough anterior chamber depth (ACD) to assist in the unfolding of the donor lenticule and to reduce the endothelial cell loss (ECL) and other adverse effects on intraocular structures. For Asian eyes with reduced ACD and high vitreous pressure there is a higher risk of lens injury, which could induce an iatrogenic cataract and require a secondary cataract surgery [7], [8], [9].

As a deep ACD is helpful for easier lenticule insertion and unfolding of the donor lenticule, some authors suggest using DSAEK to perform concomitant lens extraction on phakic eyes. [8], [10] Lenses of pediatric patients, however, may still be transparent. Reserving the transparent lens has been debated, but it has been demonstrated that retaining the lens may increase procedural risks and ECL. Other studies have shown that reserving the lens may simplify the surgical procedure, reduce disturbance of intraocular structures, and maintain accommodative function. [11] Pediatric and adult patients with phakic or pseudophakic eyes had a similar result with DSAEK [12]–[14].

To determinate outcomes and complications of DSAEK in Chinese patients, we respectively studied all Chinese patients with phakic eyes who underwent DSAEK at Peking University Third Hospital from August 2008 to August 2011.

## Patients and Methods

### Patients

Data of all patients with phakic eyes who underwent DSAEK at Peking University Third Hospital from August 2008 to August 2011 were included in the study. The study protocol and consent forms were approved by the Institutional Review Board of Peking University Third Hospital. Written informed consent was obtained from each patient or their guardian, if the patient was under 18 years of age.

General data, corneal diseases, combined ocular diseases, and previous ocular surgical history were obtained from each patient. A detailed examination was performed that included a slit-lamp exam, Heidelberg retinal tomography (HRT III, Heidelberg, GER), Visante anterior segment optical coherence tomography (AS-OCT, Carl Zeiss Meditec, Dublin, CA), and photography. Distance visual acuity (DVA), near visual acuity (NVA), intraocular pressure (IOP), ACD, central corneal thickness (CCT), and corneal endothelial cell density (ECD) were compared before DSAEK and 12 months post-DSAEK. Success rate, ECL, complications, and prognosis were analyzed. All patients had at least 12 months of follow-up visits.

### Presurgical Procedure

To provide additional protection to the lens prior to surgery, pilocarpine 2% eyedrops were administered three times preoperatively to provide additional protection of the lens.

### Surgical Procedure

Surgery was performed under general anesthesia or peribulbar anesthesia with a 50% mixture of lidocaine (2%) and bupivacaine (0.5%).

### Preparation of Plant Bed

A 5.0-mm scleral incision was made on the superior limbus. With the aid of Healon GV (Abbott Laboratories, Inc., Abbott Park, Ill, USA), the Descemet membrane and endothelium were stripped from the central area of the recipient’s cornea. The diameter of the stripped area was dependent on each patient’s specific requirements. To assist with the surgery, auxiliary incisions were made at both sides of the main incision and at the inferior limbus. A drainage incision was made from the limbus at the 4 o’clock position. Irrigation was placed in the anterior chamber. Peripheral iridectomy was routinely performed to prevent pupillary block. The position of the iridectomy was dependent on each patient’s specific conditions.

### Preparation of Donor Tissue

The donor tissue was prepared by the Moria automated lamellar therapeutic keratoplasty microkeratome equipped with a 300 micron head and associated artificial AC (Moria Inc. Doylestown, PA, US). Following the microkeratome pass, the anterior stromal “cap” was removed, and the donor button mounted on the artificial AC was brought under view of the operating microscope. The graft diameter size was dependent on each patient’s specific requirements, and ranged from 7 mm (young children) to 9 mm (patient with congenital glaucoma) with an average of diameter of 8 mm.

**Figure 1:**
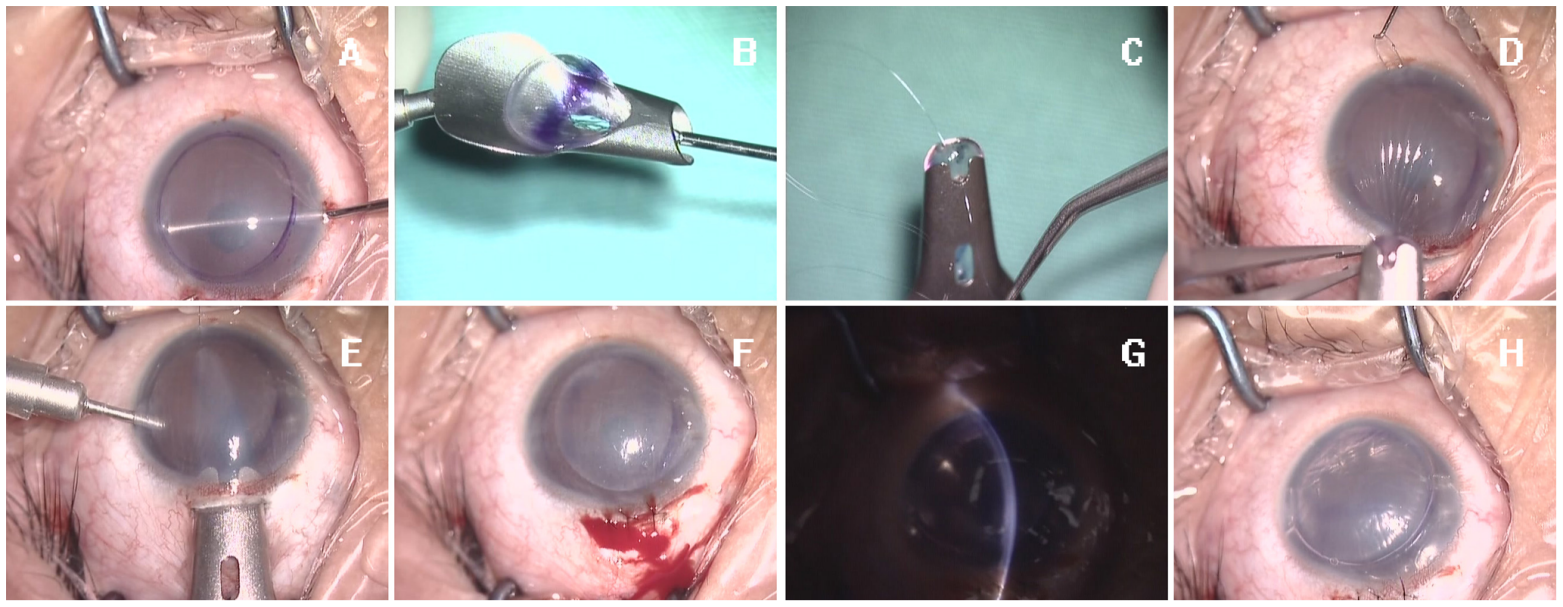
The standard DSAEK suture pull-through technique: (A) Stripping of the Descemet membrane. (B) The donor lenticule is folded into a “taco” shape with endothelial cells enclosed. The “taco” was then placed in the Busin Glide and pulled through with forceps. (C) An anchoring 10/0 prolene stitch was placed on the donor disc at the 6 o’clock position. (D) The Busin glide was brought to the main limbal incision at the 12 o’clock position. (E) The donor lenticule disc was inserted by pulling the stitch and the disc into the anterior chamber under small flow irrigation. (F) Suturing the main incision. The donor lenticule is unfolded by increased irrigation. The anchoring prolene stitch is then cut. (G) The fluid between the lenticule and plant bed is removed and a slit lamp is used to confirm good attachment. (H) A lenticule-size bubble is injected.

The donor lenticule were folded into a “taco” shape. The “taco” was then placed in the Busin glide (Moria Inc. Doylestown, PA, US) and pulled through with forceps (fig. 1). An anchoring 10/0 prolene stitch was placed on the donor lenticule at the 6 o’clock position. After the suture was passed through the tissue, it was tied to create a loop. The suture loop diameter was 4–5 mm to facilitate cutting after insertion. [2].

### Donor Lenticule Insertion

The anterior chamber was filled with Healon GV. An anchoring stitch was passed though the auxiliary incision, exiting at the limbus at the 6 o’clock position. The Healon GV in the anterior chamber was removed with a balanced saline solution (BSS, Alcon, Fort Worth, TX, USA). The Busin glide was brought to the main limbal incision at the 12 o’clock position. Using a small amount of irrigation and pulling the stitch, the donor lenticule disc was inserted into the anterior chamber. The main incision was sutured. Irrigation was increased and the donor lenticule was unfolded. The anterior chamber was sufficiently deep and well maintained. The position of the donor lenticule was adjusted to attach to the matching area of the Descement membrane stripping and the anchoring prolene stitch was cut. No instrument entered the anterior chamber during the insertion procedure. A lenticule-sized bubble was injected from the auxilliary incision. Removal of fluid between the lenticule and plant bed was performed externally by using a laser in situ keratomileusis roller device (Fig. 1). The air bubble filled the anterior chamber for 15 minutes and was reduced to 75% at the end of the surgery. The patients were placed in a face-up position on a portable hospital bed and were returned to the ward where they remained in the face-up position without a pillow for 4 hours. Young patients may need the help of their guardian.

### Postoperative Management

Topical corticosteroid (prednisolone acetate, 1.0%, Allergan Inc., Irvine, CA, USA), levofloxacin eye drops (0.5%, Santen Co. Ishikawa, Japan), cyclosporin eye drops (1%, North China Pharmaceutical Company, Ltd., Shijiazhuang, Hebei Province, China), and artificial tears were administered 4 times daily for one week. Dosage was reduced gradually as clinically indicated and was stopped 12 months post-surgery. No mydriatics were used postoperatively in case of peripheral anterior chamber occlusion.

### Statistical Analysis

SPSS 16.0 software (SPSS Inc., Chicago,Ill.,USA) was used in this study. The differences of the preoperative and postoperative data were compared by the Wilcoxon test, and P<0.05 was considered statistically significant.

## Results

One surgeon (Prof. Jing HONG) performed a total of 285 DSAEK procedures from August 2008 to August 2011. The procedures included 77 cases with phakic eyes. Twenty-one eyes of 16 patients did not undergo concomitant lens extraction because they had clear crystalline lenses and the patients were less than 50 years of age. The study included 11 males (4 patients with both eyes) and 5 females (1 patient with both eyes). Ages ranged from 2 to 47 years with an average age of 29.8 years old. The average follow up was 15.4 months (ranging from 12 to 36 months).

Diagnoses included 7 eyes (4 patients) with corneal endothelial dystrophy. Among these, there were 6 eyes (3 patients) with congenital corneal endothelial dystrophy and one eye (1 patient) with Fuchs endothelial dystrophy. Fourteen eyes (12 patients) had bullous keratopathy (BKP). Primary diseases included trauma in 2 eyes (2 patients); iris-fixated intraocular lens (IOL) in 3 eyes (2 patients); iridocorneal endothelial syndrome (ICE) in 3 eyes (3 patients); and glaucoma in 6 eyes (5 patients). There were 3 post-trabeculectomy eyes and 2 eyes with drainage valves (Table 1).

**Table 1:** Corneal Endothelial Disease.

Type	Cause	cases/eyes	age	Gender(M/F)
Corneal endothelial dystrophy	Congenital	3/6	2–7	2/1
	Fuchs	1/1	34	1/0
BKP	Glaucoma	5/6	23–43	4/1
	Trauma	2/2	31–41	2/0
	ICE	3/3	33–47	1/2
	Iris-fixated IOL	2/3	32–47	1/1

BKP: bullous keratopathy.

### Surgical Outcomes

Visual acuity data were obtained from 20 eyes of 15 patients except a 2-year-old who could not undergo a vision test. Presurgical DVA (LogMAR) was 1.7±0.7; postsurgical DVA was 0.8±0.6, *Z* = −3.517; *P*<0.001. Presurgical NVA (LogMAR) was 1.2±0.4; postsurgical NVA was 0.7±0.5, *Z* = −2.764; *P* = 0.006. The NVA of 60% of the patients (8 patients/20 eyes) was lower than 1.0 of patients who could not read prior to DSAEK. The non-assisted NVA of 70% of patients (14 patients/20 eyes) was higher than 1.0, meaning they could read following DSAEK. The non-assisted NVA of 30% of the patients (6 patients/20 eyes) was better than 0.5, meaning they could read normally without any difficulty.

**Figure 2:**
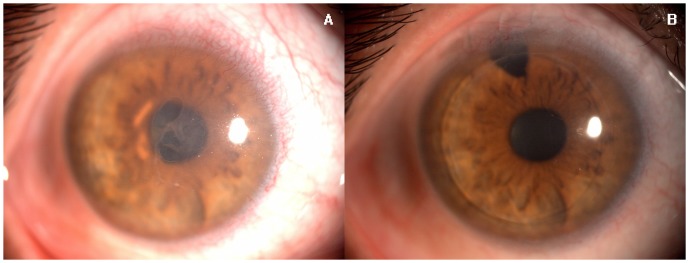
A. Bullous Keratopathy before DSAEK with a clear lens. B. Clear corneal endothelial lenticule with a clear lens 12 months after DSAEK.

**Figure 3:**
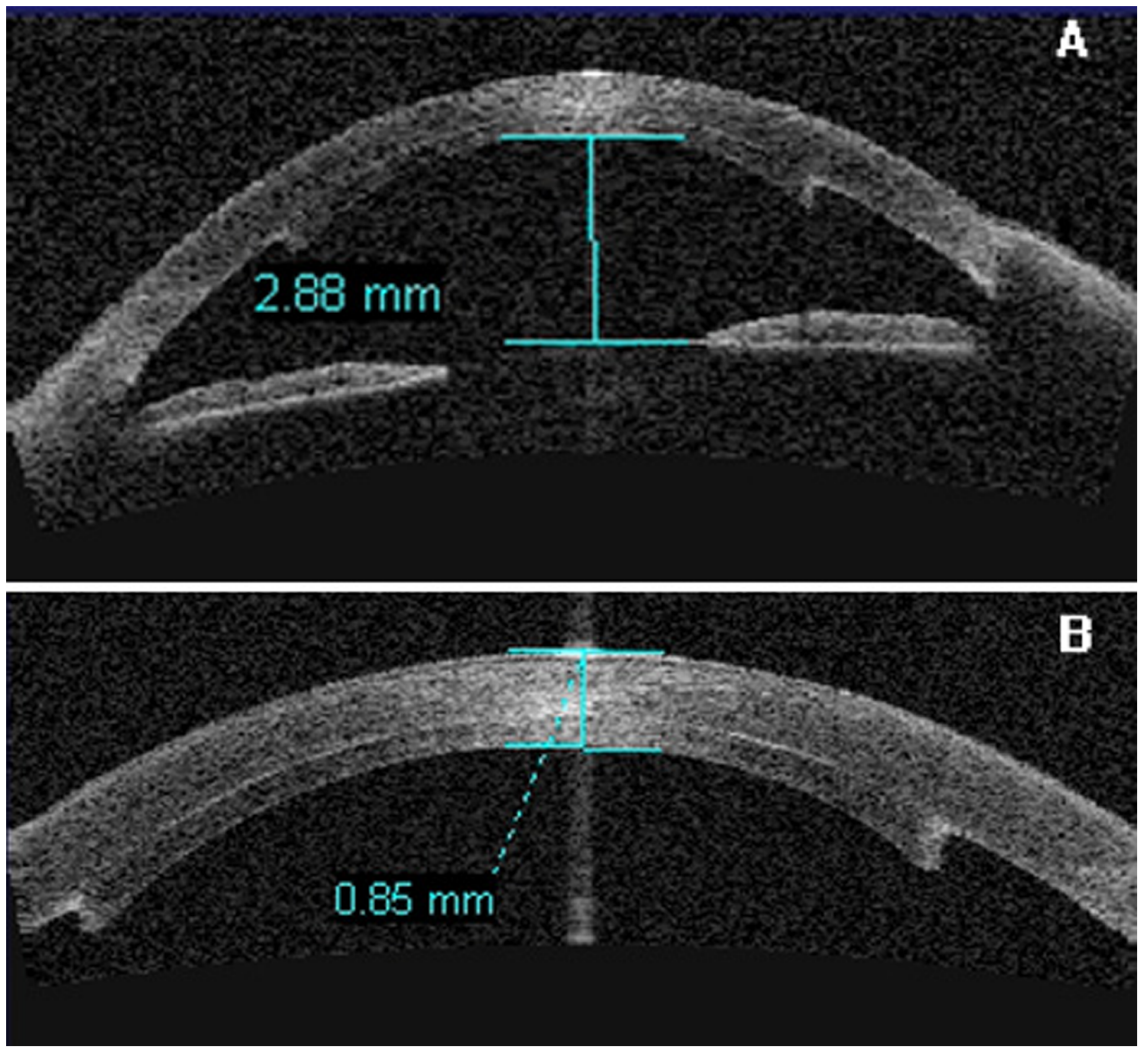
A. The ACD after DSAEK was 2.88 mm without the lenticule obstructing the anterior chamber. B. The full thickness of the cornea after DSAEK is 0.85 mm.

Presurgical IOP was 15.8±3.7 mmHg; postsurgical IOP was 15.2±2.6 mmHg, *Z* = −0.505, *P* = 0.614. Presurgical ACD was 3.00±0.74 mm; postsurgical ACD was 2.72±0.59 mm, *Z* = −0.524, *P* = 0.600. No lenticular opacities occurred during follow-up (Fig. 2, 3).

Donor ECD was 2992±163 cells/mm^2^, while 12-month postoperative ECD was 1836±412 cells/mm^2^. ECL was 39% at 12 months after DSAEK. Total corneal thickness was 0.78±0.18 (0.54–1.08) mm before DSAEK and 0.74±0.07 (0.65–0.85) mm after DSAEK with receptor bed thickness of 0.55±0.08 (0.47–0.70) mm and donor lenticule thickness of 0.19±0.05 (0.10–0.33) mm.

At the end of the 12-month follow up, the donor graft survived in 18 eyes; dysfunction or change occurred in 3 eyes. The surgical success rate was 86%.

### Surgical Complications

Surgical complications were encountered in 9 cases (Table 2). The most common complication was pupillary block with markedly elevated IOP, which occurred in 5 eyes (24%). All complications occurred during the first postoperative night. The IOP of 2 eyes increased to approximately 30 mmHg, decreasing to normal following antiglaucoma oral medicine and treatment with eyedrops. During the follow-up period, the lenticules remained transparent. Three eyes had IOP as high as 60 mmHg, and part of a bubble in the anterior chamber was removed. One lenticule became transparent while the other 2 had consistent edema (longer than 7 days after DSAEK) and ultimately loss function. Of the two eyes, one received a lenticule exchange 10 days after the first DSAEK procedure. With fewer gas bubbles, there was no elevation of IOP, and the lenticule retained transparency during follow up.

**Table 2:** Surgical Complications.

No	Age	Diagnosis	Complication	Treatment	Prognosis
1	33	BKP	Hypertension	Antiglaucoma medicine	Success
2	32	BKP	Hypertension	Antiglaucoma medicine	Success
		Iris-fixated IOL			
3	22	BKP	Hypertension	Bubble releasing	Success
		Glaucoma			
4	47	BKP	Hypertension	Bubble releasing	Graft exchange
		Iris-fixated IOL			
5	41	BKP	Hypertension	Bubble releasing	Graft dysfunction
		Trauma			
6	3	Congenital endothelial dystrophy	Graft detachment	Graft reposition	Success
7	2	Congenital endothelial dystrophy	Graft detachment	Graft reposition	Success
8	2	Congenital endothelial dystrophy	Graft detachment	Graft reposition	PKP
9	43	BKP	Graft dislocation	Graft reposition	Success
		Glaucoma			

BKP: Bullous Keratoplasty, PKP: Penetrating Keratoplasty.

Graft detachment occurred in 3 eyes of 2 pediatric patients (14%). We performed surgery to reposition and reattach the two lenticules and again injected air bubbles. The lenticules were transparent during the follow-up period. Due to the young age of the patient (2-years-old), one lenticule was difficult to reposition, so PKP was performed one month after DSAEK.

Graft dislocation occurred in 1 eye (5%) and a repositioning was performed the next morning. The lenticule was transparent during the follow-up period. No cataract, acute rejection, or primary graft failure [3] occurred in our study.

## Discussion

The success of DSAEK depends on the postoperative ECD. It has been reported that ECL at 1 year after DSAEK was 24–61%. [3], [11] Surgical manipulation is important for donor endothelial cell survival. The deeper anterior chamber facilitates surgical manipulation and supports donor endothelial cell preservation. Therefore, some authors suggest removing the clear crystalline lens to obtain a larger anterior chamber space when performing DSAEK. [8].

In the early stages of DSAEK, the lens damage rate was higher due to a thicker donor lenticule and instruments having to enter the anterior chamber multiple times. [8], [15] The development of the suture pull-through insertion technique minimizes the need of surgical instruments having to enter the anterior chamber. [16]–[18] The insertion method we use can prevent the instrument from reaching the pupil. The iris acted as a barrier to the instrument touching the crystalline lens. The donor lenticule was pulled into the anterior chamber by an anchoring stitch, so there was no mechanical damage to donor endothelial cells and the crystalline lens. In addition, during the insertion procedure, the closed anterior chamber may provide enough IOP to contend with vitreous pressure. We believe this insertion method, which prevents mechanical damage to the donor lenticule or crystalline lens, may benefit patients with phakic eyes. Therefore, we used the DSAEK suture pull-through insertion technique for young patients who need to retain their crystalline lens.

### Patient Characteristics

All patients in our study were younger than 50 years of age; 56% of patients (9 patients/16 eyes) were younger than 35 years of age; and 3 patients (6 eyes) were under 10 years of age. All of the patients’ lenses were transparent prior to DSAEK. We think it is not suitable to remove the clear crystalline lens of children and adults with normal accommodative function, because such function is important for reading and near-distance activities of daily life. Therefore, we performed a simple DSAEK procedure and preserved the lens.

### Surgical Outcomes

The DVA and NVA improved significantly after surgery (*P*<0.001,*P* = 0.006, respectively). Other than a 2-year-old patient who could not undergo a vision test, 60% of patients (8/20) could not read before DSAEK, while 70% (14/20) could read, and 30% (6/20) were able to achieve a normal reading speed and reading endurance. [19] Our study demonstrated that by retaining the crystalline lens patients could obtain improved NVA without assistance, and even normal NVA. Preservation of accommodative function is extremely important, especially for younger patients and children, as it enables consistent distance, middle-distance, and near VA. Futher, it may help to improve reading speed, reading endurance, and improve quality of life. Research has shown this technique can be safely and effectively applied to children and may potentially avoid amblyopia. [13] Visual acuity was measured in our study 12 months after DSAEK. We are optimistic that pediatric patients with amblyopia will achieve better long-term VA.

The IOP of our patients did not increase at the 12-month follow up (*P* = 0.614). The ACD did not decrease (*P* = 0.600). Total corneal thickness was 0.78±0.18 (0.54–1.08) mm before DSAEK and 0.74±0.07 (0.65–0.85) mm after DSAEK with receptor bed thickness of 0.55±0.08 (0.47–0.70) mm and donor lenticule thickness 0.19±0.05 (0.10–0.33) mm. AS-OCT showed that the lenticule did not block the anterior chamber angle. The ECL was 39% at the 12-month follow up, less than the 42% shown in other studies. [3] At the end of the 12-month follow up, the donor graft survived in 18 eyes; dysfunction or change occurred in 3 eyes. The surgical success rate was 86%. This may imply there is no difference in ECL, whether or not the clear crystalline lens is reserved. Progressive insertion techniques may have good outcomes on DSAEK with phakic eyes.

### Surgical Complications

#### Pupillary block hypertension

There were 9 cases of surgical complications in our study. The most common complication was pupillary block with markedly elevated IOP, which occurred in 5 eyes (24%) and was higher than the previous study (3%). [3] All complications occurred the first night after surgery. Three lenticules (60%) became transparent while the other 2 (40%) lost function. This complication may be due to the smaller anterior chamber space of Chinese patients. The average ACD before DSAEK was 3.00 mm. We had performed peripheral iridectomy, but large amounts of bubbles in the anterior chamber caused pupillary block and increased IOP. The 2 cases in which the lenticules lost function were both complicated bullous keratopathy cases. One case was secondary to trauma, and the other complication was an iris-fixated IOL in the anterior chamber. We hypothesize the complicated intraocular structure may induce severe hypertension secondary to pupillary block. The donor endothelial cell loss was more significant and caused dysfunction.

It is important to choose the appropriate bubble size for patients with phakic eyes. A bubble size larger than the lenticule may facilitate the attachment but may more easily induce pupillary block. We recommend a bubble size equal with the size of the donor lenticule. The bubble should be released as soon as possible when uncontrolled hypertension occurs postoperatively so that there is less corneal endothelial cell loss.

#### Other complications

Graft detachment occurred in 3 eyes of 2 patients (14%). This was less than Busin *et al*, who reported 25% in phakic pediatric patients [13] but was similar to the meta analysis data of Lee *et al* who also reported graft detachments of 14%. [3] In our study we had 2 pediatric patients requiring PKP one month after DSAEK to reattach a lenticule. We hypothesize that risk factors for postoperative graft detachment in pediatric patients include their difficulty in maintaining a supine position and the small bubble used to prevent pupillary block.

Another rare complication found in our study was graft dislocation (5%). The patient’s primary disease was congenital glaucoma with macrocornea and irregular corneal curvature. We chose a 9 mm donor lenticule, but graft dislocation occurred the next morning. We repositioned the lenticule and injected more bubbles, and the lenticule remained attached and transparent during the follow-up period. In our study, there was no occurrence of cataract, acute rejection, or primary graft failure [3].

Our research indicates few previous studies regarding DSAEK in phakic eyes. These studies had a small sample size (Table 3), and patients were primarily adults [8], [11], [12], while the patients in our study ranged from 2 to 47 years of age. The other marked difference between previous studies and our study was the types of corneal endothelial disease encountered. The patients in previous studies had Fuchs endothelial dystrophy or drug-induced endothelial cell dysfunction without anterior chamber changes. [8], [11]–[13] The patients in our study had more complicated diseases; primary disorders included congenital diseases, trauma, glaucoma, and iris-fixated anterior chamber intraocular lens. The patients’ lenses were clear and were reserved. We used the DSAEK suture pull-through insertion technique and achieved a good outcome. The ECL and incidence of complications were similar with previous studies [8], [11]–[13].

**Table 3:** Current Studies about DSAEK on Phakic Eyes.

Year/Author	Eyes/Patients	Average age(Range)	Diagnosis	Insertion methods	Followup (M)	ECL	Complications
2010 Koenig S	6/4	49(40–60)	Fuchs/drug toxicity	Forceps	9(6–14)	−	2 graft dysfunction
2011 Huang T	6/5	46(38–53)	Fuchs	−	12–27	31.6%*	1 mild cataract
2011 Tsui J	10/10	55(43–68)	Fuchs	Forceps/Cartridge Injector	18–24	58%* †	4 cataract2 graft detachment1 graft rejection
2011 Busin M	15/8	9(0.5–30)	Fuchs	Busin Glide	15.9(3–48)	30%	4 graft detachment
2012 Our study	21/16	29.8(2–47)	Fuchs/BKP	Suture auxilliary Busin Glide	15.4(12–36)	39%*	5 hypertension3 graft detachment1 graft dislocation

* ECL at 12 months postoperatively,

† including 2 eyes that underwent cataract surgery.

The limitation of our study was a relatively small sample size, however, this may be the largest sample size to date. Future studies are warranted to consider risk factors related to different corneal diseases. Long-term patient follow up is also needed.

Our study indicated DSAEK on Chinese phakic eyes can significantly improve DVA and NVA by preserving the patient’s own crystalline lens. DSAEK is an optional surgery for patients who need to preserve accommodative function. More attention should be given to postsurgical pupillary block-induced hypertension.
